# PDE-5 Inhibitors in Scleroderma Raynaud Phenomenon and Digital Ulcers: Current Status of Clinical Trials

**DOI:** 10.1155/2011/392542

**Published:** 2011-11-02

**Authors:** Ann J. Impens, Kristine Phillips, Elena Schiopu

**Affiliations:** University of Michigan Scleroderma Program, Ann Arbor, MI 48106-5753, USA

## Abstract

Systemic sclerosis- (SSc-) related vasculopathy, as manifested by Raynaud's Phenomenon (RP) and digital ulcers (DUs), is associated with significant impairment of the quality of life and morbidity. The current vasoactive approach for SSc-RP, although employing vasodilators, is entirely off-label. PDE-5 inhibitors improve peripheral circulation, are well tolerated, and are widely used for various forms of constrictive vasculopathies. This class of medications has become one of the first lines of treatment of SSc-RP and SSc-DUs among rheumatologists that routinely treat SSc patients. Due to the lack of robust randomized clinical trials of PDE-5 inhibitors in SSc-RP/DUs, the PDE-5 inhibitors have not been FDA approved for these particular indications, which constitutes a significant barrier to prescribing this category of drugs. This paper reviews the current state of evidence-based knowledge in SSc-related vasculopathy and the use of PDE-5 inhibitors.

## 1. Introduction

Phosphodiesterases (PDEs) are isoenzymes that control the level of intracellular cyclic guanosine monophosphate (cGMP) and cyclic adenosine monophosphate (cAMP) by hydrolyzing them [1]. The human genome encodes 21 PDE genes which are classified in 11 families. PDE isoenzyme 5 (PDE-5) selectively breaks down the cGMP, a critical smooth muscle tone regulator. Nitric oxide (NO), produced by nitric oxide synthase, signals the conversion of GMP into cGMP which accumulates inside the cell. Inhibition of the PDE-5 enzyme increases the available intracellular cGMP which leads to vasodilatation. Aside from corpus cavernosum, PDE-5 is found on a variety of tissues, including platelets, lungs, muscle, brain, retina, thymus, heart, liver, esophagus, stomach, pancreas, small intestine [1], arterial and venous vasculature [2], and endothelial cells [3]. 

**Figure 1:**
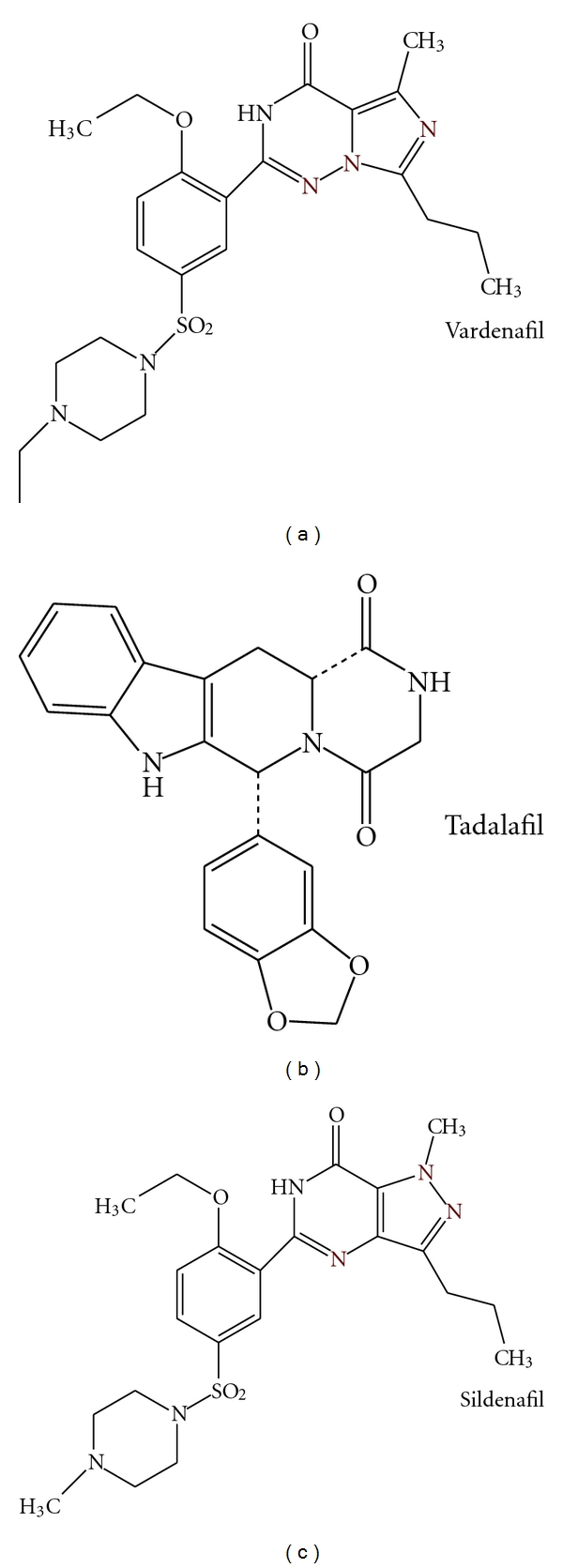
Chemical structures of the three available PDE-5Is [4].

Sildenafil, vardenafil, and tadalafil are the three commercially available PDE-5 inhibitors (PDE-5Is). All three PDE-5Is are available in oral formulation, are rapidly absorbed from the gastrointestinal tract, and are metabolized by hepatic enzymes via cytochrome P450 [4]. Sildenafil and vardenafil have similar molecular structures, while the tadalafil molecule is different, the difference being reflected in the pharmacokinetic properties (Figure 1) [4]. Tadalafil is not affected by food ingestion and has a terminal half-life of 17.5 hours as opposed to sildenafil and vardenafil which are affected by fatty food intake and both have a half-life of approximately 4 hours [4].

The primary Food and Drug Administration- (FDA-) approved indication for the PDE-5Is is erectile dysfunction. In recent years, sildenafil (2005) [5] and tadalafil (2009) [6] have also been approved for use in pulmonary arterial hypertension. Vardenafil was recently shown to improve hemodynamic parameters in patients with pulmonary arterial hypertension in a randomized trial of 66 patients [7].

Raynaud's Phenomenon (RP) is an exaggerated vasoconstrictive response to cold and stress and is the presenting symptom in the majority of patients with systemic sclerosis (SSc) [8]. An important clinical manifestation of the scleroderma-related vasculopathy is the ischemic digital ulcer (DU) which is associated with significant morbidity [9]. Use of PDE-5Is in SSc-related RP and DU makes pathophysiologic sense and has been explored in randomized fashion.

## 2. Clinical Trials

As with penicillin or TNF-*α* blockers, the PDE-5Is history is interesting. The initial intent was to develop PDE-5Is as a new anti-ischemic therapy, but the early cardiac trials failed to excite any interest. The “adverse” effect on penile erections led to revolutionary development of erectile dysfunction awareness and therapies [10]. Sildenafil citrate (Viagra) was approved by the FDA in 1998. Another PDE-5I, vardenafil (Levitra), came to market in September of 2003, followed shortly by the “weekend” drug, tadalafil (Cialis), in November of 2003. Each of these individual drugs' use in SSc-RP and DU will be reviewed below.

### 2.1. Sildenafil

The popular “blue pill” for erectile dysfunction has been used off-label by rheumatologists for symptomatic improvement of secondary RP and SSc-DUs. A retrospective chart review of 10 SSc patients at a single center briefly described the response to sildenafil dosed from 12.5 mg to 100 mg daily [11]. As the letter to the editor reports in 2005, “eight of the ten patients [⋯] had a response within few weeks, with significant reduction in the frequency and severity of RP. Of the eight patients who had digital ulcers [⋯] six experienced complete healing of the ulcers.” No other details were provided regarding the specific measures used to quantify the RP improvement [11]. 

The physiological benefit of sildenafil citrate in patients with SSc-RP was assessed in a group of 5 patients and published as a letter to the editor in 2006 [12]. In this small study, the objective measure of the skin temperature response to mild cold challenge after a single dose of 50 mg of sildenafil citrate was conducted by thermography (thermal images of the hands were collected every minute for 15 minutes after the cold challenge to enable an area under the curve) and by percentage recovery to mean baseline temperature postcold challenge. Although this letter reported that 3 out of the 5 patients had clear and significant improvement in digital temperature responses to mild cold challenge, no other details were provided [12]. Based on this limited information, sildenafil citrate seems to be well tolerated in patients with SSc-RP.

A randomized double-blind cross-over trial of single dose of 50 mg sildenafil or *α*-tocopherol (100 mg) was reported in abstract form in 15 patients with RP [13]. The outcome measures reported were 75% improvement in forearm blood flow (as measured by near-infrared time-resolved spectroscopy) and 32% increase in serum cGMP concentration (*P* < 0.01) in the sildenafil group. Details about how many patients had SSc-RP are not available. The study reported no adverse events, aside from a mild decrease in the systolic and diastolic blood pressure (*P* < 0.05) in the patients exposed to sildenafil [13]. The only published trial that evaluated efficacy of sildenafil in RP is a double-blinded, placebo-controlled, fixed-dose, cross-over study of 50 mg sildenafil twice daily for 4 weeks versus placebo [14]. Most subjects had secondary RP (16/18), and 6 of the patients with secondary RP had DUs. Primary outcome variables included RP frequency and duration as assessed by diary cards, Raynaud's Condition Score (RCS), capillary flow velocity by laser Doppler anemometry, and healing of the DUs. At the end of the study, there was significant improvement in the frequency (35 versus 52, *P* = 0.0064) and duration (581 versus 1046 minutes, *P* = 0.0038) of RP attacks and in the RCS (2.2 versus 3.0, *P* = 0.0386). After 4 weeks of active therapy with sildenafil, 2 of the 6 subjects with DUs completely healed their ulcers, while the rest of the subjects noted visible healing. The mean capillary blood flow velocity increased by more than 400% (from 0.13 to 0.53, *P* = 0.0004) during the sildenafil treatment [14]. Despite its small size, this trial confidently shows improvement in the peripheral circulation after exposure to sildenafil and improvement of traditional measures of RP.

Aside from case reports, the effect of sildenafil on SSc-DUs was not systematically studied. In 2010, Brueckner et al. reported the results of a pilot open-label study of the effects of maximum tolerated sildenafil in SSc-DUs [15]. Sixteen patients were treated with a mean sildenafil dose of 114 mg/day for a mean duration of 5.2 months. There was significant improvement (*P* < 0.001) in the number of DUs from baseline (mean of 3.1/patient) to the end of sildenafil therapy (mean of 1.1/patient). Most patients reached a minimum number of DUs within 3 months irrespective of SSc. Nine subjects developed a total of 12 new DUs while taking sildenafil, of which one of the subjects was diagnosed with calcinosis [15]. Based on this small, open label trial, it seems that sildenafil is well tolerated, and it could be a viable option for SSc-DU therapy. An important caveat of this study is the lack of uniformity in defining SSc-DU (DUs due to calcinosis tend to respond less to vasoactive therapies) and the absence of a control group.

### 2.2. Vardenafil

Vardenafil is similar to sildenafil in terms of pharmacokinetic properties, with rapid onset of action, maximal benefit at 1 hour, and a half-life of about 4 hours. It is the second PDE-5I to make its debut in the erectile dysfunction arena at a dose of 10 mg as needed. 

Caglayan et al. published the results of an open-label pilot study of vardenafil (10 mg twice daily for 2 weeks) in patients with RP in 2006 [16]. Of the 40 subjects recruited, 33 (82%) had secondary RP. All the vasoactive medications were discontinued at least one week prior to recruitment, and the outcome measures included RCS, frequency, and duration of RP attacks, and measures of peripheral blood flow by laser Doppler's flowmetry at room temperature and in the cold exposure test room. Laser Doppler's flowmetry revealed that 70% (28) of the subjects had improved digital flow, and, in those individuals, the digital blood flow measured at room temperature increased by a mean of 21% and 30% at 1 hour and 2 weeks compared to baseline at (*P* < 0.01). The RCS improved significantly from baseline to the second week of vardenafil therapy (5.05 versus 3.54, *P* < 0.001) [16]. As far as we could ascertain this is the only published trial of vardenifil. Based on this information vardenafil seems to be well tolerated and shows RP improvement. 

### 2.3. Tadalafil

The longer half-life of this PDE-5I made tadalafil an attractive option as a daily dose for SSc-related peripheral vasculopathy. A small open-label study of tadalafil (between 5 and 20 mg every other day as needed) was published in abstract format in 2005 [17]. Of the 15 patients studied, 11 had SSc spectrum of diseases and the RCS was reported to be 3.8 while on tadalafil versus 6.9 without. Due to the limited data available in the abstract, no meaningful conclusions can be derived from this study.

A physiological study of tadalafil in RP (mostly primary RP, 18/20) was reported in 2007: this was a double-blind, placebo-controlled cross-over study of 20 patients with RP that received a single dose of 10 mg of tadalafil versus placebo [18]. The hypothesis that tadalafil improves cold-induced vasoconstriction was tested by measuring the digital blood flow with laser Doppler flowmetry at rest and during two graduated local heat and cold exposure cycles; skin blood flow (flux) and skin temperature were recorded at baseline and 90 minutes after receiving the drug. Tadalafil did not affect the baseline flux (*P* = 0.57) or skin temperature (*P* = 0.69). Tadalafil neither increased the maximal flux flow during heating nor decreased the vasoconstriction during cooling, which might mean that tadalafil improves RP through a different mechanism [18]. 

There is evidence that tadalafil improves peripheral circulation in SSc-RP. A randomized controlled trial of tadalafil (20 mg 2-3 times/week) versus pentoxifylline for 4 weeks in men with severe RP associated with autoimmune diseases was reported in abstract form in 2006 [19]. The frequency (decline of 59% with tadalafil versus 36% with pentoxifylline) and duration of RP attacks and the RCS were improved in the patients receiving tadalafil. Also, physician and patient assessments of RP improved at 4 weeks (*P* < 0.05 compared to 2 weeks, and *P* < 0.05 versus controls). Our interpretation of this study is limited as no specific information is provided about the number of SSc patients included in this study or about the statistical significance of some of the results. A more recent open-label study of 20 male patients with SSc-RP receiving 10 mg of daily tadalafil for 12 weeks was published in 2009. The primary endpoint showed improvement in the RCS, the number of RP attacks and a decrease in the plasma adrenomedullin and endothelin-1 levels compared to baseline [20].

In 2009, Schiopu et al. reported a randomized, double-blinded, placebo-controlled, crossover trial of tadalafil at a fixed dose of 20 mg daily versus placebo in 39 women with SSc-RP [21]. The trial design prohibited use of any other vasodilator therapies for RP, required a run-in period to document presence of a minimum of 6 RP attacks per week and excluded smokers. The treatment blocks were 4 weeks each with a 2-week washout in between. The outcome measures included paper RP diary and RCS. There were not sufficient DUs to permit an adequate statistical analysis of the effects on DUs. Although all measures showed overall improvement, there was no significant difference between the change from baseline RCS, duration, and frequency of attacks among the tadalafil and the placebo groups (RCS 2.43 versus 2.53, frequency 2.08 versus 2.1, duration 40.61 versus 47.0) [21]. A year later, Shenoy et al. described the results of a single-center, randomized, double-blind cross-over trial of tadalafil at 20 mg on alternate days versus placebo in subjects with SSc-RP which was conducted in India [22]. The trial design, although similar to the previous cross-over trial of tadalafil, included a longer treatment block (6 weeks) and a shorter washout (1 week), and allowed subjects to continue all the previous RP-specific therapies. When compared to the Schiopu et al. trial, the patients recruited needed to have 4 RP attacks/week, as opposed to 6, and the mean age of the 24 participants was younger (36.87 versus 52.9). The primary outcomes were similar: mean change in RCS, duration, and frequency of RP attacks from baseline. The secondary outcomes included assessment of DUs, quality of life (QoL) measures, endothelial function, and flow-mediated dilatation of brachial artery. In this study, the primary outcomes were significantly better in the tadalafil group: frequency (2.29 versus 3.37, *P* < 0.001), duration (33.81 versus 54.89, *P* = 0.023), and RCS (3.86 versus 5.20, *P* < 0.0005). All the 24 digital lesions healed during the tadalafil treatment versus only 3 of the 13 DUs during the placebo treatment. The brachial artery reactivity was measured using B-mode ultrasound imaging and improved significantly while subjects received active drug (*P* < 0.05). The levels of E-selectin and endothelin 1 were not significantly different between placebo and the active treatment groups (*P* = 0.5 and 0.81, resp.) [22].

Following this above-mentioned single-center trial by Shenoy et al, a multicenter randomized, double-blinded, placebo-controlled study of tadalafil at 20 mg every other day versus placebo was conducted in 4 centers from North and Northeastern India and reported in an abstract form [23]. Similar to Shenoy et al., the mean age of the recruited subjects was lower than the mean age of the subjects in the US tadalafil trial (36.8 years in both Indian studies versus 52.9 years in the US study). This trial recruited 53 subjects, and, after a 2 week run-in period, they were randomized to tadalafil 20 mg every other day or placebo for the 8-week treatment period. Similar to the Shenoy et al. trial, improvement in RCS, duration, and frequency of RP attacks was demonstrated (*P* < 0.05, *P* < 0.001, *P* < 0.001, resp.), along with significant SSc-DU healing (14 out of 18 DUs healed in the tadalafil group compared to 5 out of 13 in the placebo group) [23]. It is likely that the differences in the trial design, specifically the concomitant RP therapies, along with the lower average age (less disease severity and more reversal changes, consistent with younger age) could explain the differences in the results of these controlled trials. A significant aspect on which all three studies seem to agree was the excellent tolerability of the oral tadalafil as a daily or every other day therapy for SSc-RP.

### 2.4. Ongoing Clinical Trials

A phase IIa, randomized double-blinded, placebo-controlled, cross-over trial to assess the efficacy of a novel, once daily PDE-5I (PF-00489791) for the treatment of primary and SSc-related secondary RP has just finished recruiting. This is a multicenter trial sponsored by Pfizer (http://www.clinicaltrials.gov/ identifier NT01090492). Another phase II/III clinical trial is comparing daily use of amlodipine (10 mg) versus udenafil daily (100 mg) in secondary RP in a double-blind, randomized, cross-over design; aside from the RP common outcome measures, the investigators are also assessing DUs and arterial flow velocity (http://www.clinicaltrials.gov/ identifier: NCT01280266).

The effects of sildenafil in SSc-RP as measured by the microcirculator blood flow, endothelial progenitor cells, and serum levels of vascular endothelium growth factor is ongoing (http://www.clinicaltrials.gov/ identifier: NCT01347008). A multicenter double-blinded, placebo-controlled trial is also currently randomizing participants to either sildenafil (20 mg three times a day) or placebo for 90 days to assess healing of SSc-DUs (http://www.clinicaltrials.gov/ identifier: NCT01295736).

## 3. Conclusions

The SSc-related vasculopathy (including RP and DUs) lacks FDA-approved therapies. The class of PDE-5Is is a well-tolerated vasoactive therapy for patients with SSc-related vasculopathy. After a surge of clinical case reports, case series and open-label studies published in the mid 2000s, large, multicenter, controlled trials of PDE-5Is in SSc-related vasculopathy remain underdeveloped. Most of the published clinical trials in SSc-RP focus on sildenafil and tadalafil although the one study reported with vardenafil has shown it to be well tolerated with a beneficial impact on RP.

Currently, the available evidence for the efficacy of PDE-5I's for SSc RP/DUs is thin. Definite conclusions are hard to reach as studies have shown conflicting results. Comparisons are difficult as patient populations and outcome measures are not uniform. There are efforts to address the questions of placebo response in RP by designing trials focused on objective vascular markers, QoL, and physicians' and patients' global assessments. Robust clinical evidence that PDE-5Is, alone or in conjunction with traditional vasoactive therapy, improve SSc-RP and heal SSc-DUs is still lacking. Clinical trials involving selective PDE-5Is are ongoing.

The great clinical need for a tolerable, affordable therapeutic option for SSc-RP and SSc-DU remains. The clinical research community recognizes PDE-5Is as an excellent potential option for SSc-related vasculopathy.
